# Effect of Heat Treatment Temperature on the Crystallization Behavior and Microstructural Evolution of Amorphous NbCo_1.1_Sn

**DOI:** 10.1021/acsami.3c10298

**Published:** 2023-09-22

**Authors:** Chanwon Jung, Siyuan Zhang, Kyuseon Jang, Ningyan Cheng, Christina Scheu, Seong-Hoon Yi, Pyuck-Pa Choi

**Affiliations:** † Department of Materials Science and Engineering, Korea Advanced Institute of Science and Technology (KAIST), 291 Daehak-ro, Yuseong-gu, Daejeon 34141, Republic of Korea; ‡ Max-Planck-Institut für Eisenforschung, Max-Planck-Straße 1, Düsseldorf 40237, Germany; § Department of Materials Science and Metallurgical Engineering, Kyungpook National University, 80 Daehakro, Daegu 41566, Republic of Korea

**Keywords:** amorphous, crystallization, half-Heusler compounds, diffusion, atom probe tomography, transmission electron microscopy

## Abstract

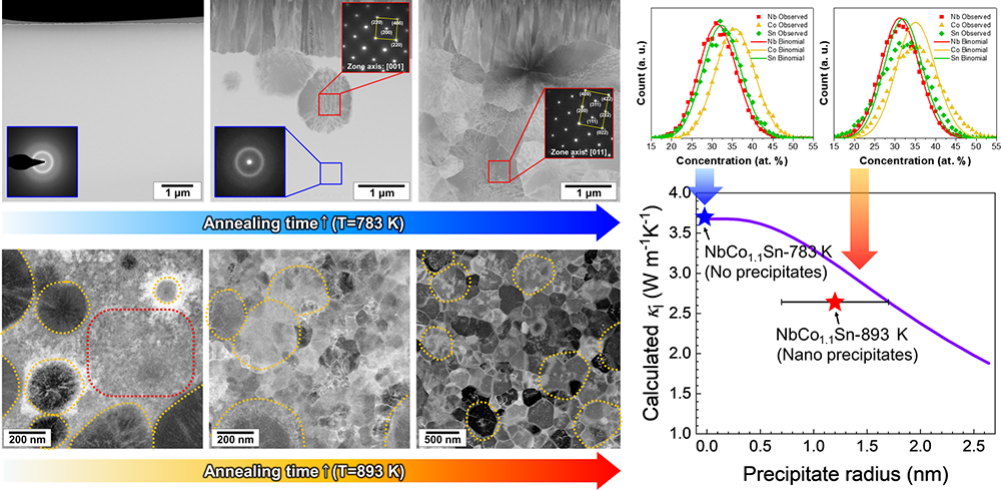

Heat treatment-induced nanocrystallization of amorphous precursors is a promising method for nanostructuring half-Heusler compounds as it holds significant potential in the fabrication of intricate and customizable nanostructured materials. To fully exploit these advantages, a comprehensive understanding of the crystallization behavior of amorphous precursors under different crystallization conditions is crucial. In this study, we investigated the crystallization behavior of the amorphous NbCo_1.1_Sn alloy at elevated temperatures (783 and 893 K) using transmission electron microscopy and atom probe tomography. As a result, heat treatment at 893 K resulted in a significantly finer grain structure than heat treatment at 783 K owing to the higher nucleation rate at 893 K. At both temperatures, the predominant phase was a half-Heusler phase, whereas the Heusler phase, associated with Co diffusion, was exclusively observed at the specimen annealed at 893 K. The Debye–Callaway model supports that the lower lattice thermal conductivity of NbCo_1.1_Sn annealed at 893 K is primarily attributed to the formation of Heusler nanoprecipitates rather than a finer grain size. The experimental findings of this study provide valuable insights into the nanocrystallization of amorphous alloys for enhancing thermoelectric properties.

## 1 Introduction

Thermoelectric power generation holds great promise for sustainable energy production because it enables the conversion of waste heat into electrical energy.^1−8^ Among the wide range of thermoelectric materials, half-Heusler compounds have obtained significant attention owing to their large power factor,^9−12^ good thermal stability,^13^ mechanical robustness,^14^ and nontoxicity^15^ and the utilization of earth-abundant elements.^16^ Additionally, half-Heusler compounds exhibit peak figure of merit (*zT*) values within the temperature range of 800–1200 K,^9^ at which a significant amount of industrial waste heat can be utilized and recycled.^17^

The figure of merit, *zT*, is a key metric for evaluating the performance of thermoelectric materials. It is defined as *zT* = *S*^*2*^σ*T*(κ_*L*_ + κ_*e*_)^−1^, where *S* represents the Seebeck coefficient, σ denotes the electrical conductivity, *T* is the absolute temperature, and κ_*L*_ and κ_*e*_ represent the lattice thermal conductivity and electronic thermal conductivity, respectively.^1^ To attain a high *zT*, high electrical conductivity (σ), high Seebeck coefficient (*S*), and low thermal conductivity (κ) are required. For half-Heusler compounds, although they exhibit high electrical conductivity and a large Seebeck coefficient, the achievement of high *zT* values is hindered by their high thermal conductivity.^18,19^

Nanostructuring has been considered the most effective approach to overcome this limitation since grain boundaries and phase boundaries act as effective scattering centers for phonons, thereby reducing the lattice thermal conductivity while minimally impacting the electrical conductivity.^20−22^ Mechanical alloying followed by sintering is a widely employed method for the fabricating of nanostructured thermoelectric materials owing to its convenience and effectiveness to reduce the lattice thermal conductivity.^23−27^ Numerous studies have reported enhanced figure of merits attributed to the reduction in lattice thermal conductivity by using this approach.^28−30^ However, undesired secondary phases or micro- and nanosegregation can occur during the sintering process, which hampers the fine control of microstructures of materials.^31,32^ Furthermore, oxidation or impurity contamination during ball milling and sintering processes can elevate lattice thermal conductivity and degrade the Seebeck coefficient, thereby resulting in deterioration of the thermoelectric properties.^32−36^ Moreover, the reduction in grain size is constrained as grain growth takes place during the consolidation process.^20^ For instance, Joshi et al. reported that the initial grain size of Hf_0.75_Zr_0.25_NiSn_0.99_Sb_0.01_ milled powder, approximately 50 nm, increased to over 200 nm after the hot-pressing process.^37^

In this regard, nanocrystallization of amorphous precursors through heat treatment shows particular promise for fabricating high-quality nanostructured materials in a scalable manner.^38−40^ This method enables the production of a highly homogeneous amorphous alloy without chemical segregation. By modifying the heat treatment conditions^41^ or adjusting the supersaturated amorphous composition,^42^ it enables to design the final microstructure with greater flexibility. This approach finds widespread application in the nanostructuring of soft magnetic materials, allowing the production of fine grain sizes as small as 10 nm while keeping an amorphous phase remaining at the grain boundaries.^43−45^ Overall, the use of amorphous precursors for fabricating nanocrystalline structures provides a pathway to obtain materials with the desired properties, improved performance, and expanded application possibilities. To fully exploit these advantages, a comprehensive understanding of the crystallization behavior of amorphous precursors under different crystallization conditions is crucial as it significantly influences the thermoelectric properties of the resulting materials.^46^

In our previous study, we fabricated two distinct microstructures with different electron and phonon transport properties by annealing amorphous NbCo_1.1_Sn at different temperatures.^38^ We revealed that excess Co leads to a NbCo_2_Sn Heusler phase, which is the primary reason for the enhanced thermoelectric properties; therefore, understanding Co diffusion and the consequent formation of Heusler compounds during the annealing is the key solution for obtaining promising nanostructures with enhanced thermoelectric properties.

In this study, we conducted a microstructure investigation of the crystallization behavior of amorphous NbCo_1.1_Sn at two different heat treatment temperatures (783 and 893 K). The grain size and phase configuration were substantially different depending on the temperature, with an observation of finer grain size obtained from the heat treatment at higher temperatures. By jointly using atom probe tomography (APT) and transmission electron microscopy (TEM), we elucidated the microstructural evolution during the heat treatment and its underlying mechanisms based on the difference of Co diffusion kinetics. As a result, the Debye–Callaway model affords the insight that Co diffusion affects the low lattice thermal conductivity of NbCo_1.1_Sn annealed at 893 K through the formation of Heusler nanoprecipitates.

## 2 Results

### 2.1 Phase Evolution during the Heat Treatment

Based on the DSC experiment conducted on the amorphous NbCo_1.1_Sn alloy using a scan rate of 40 K/min, the observed onset and peak temperatures of crystallization were 760 K (*T*_x_) and 842 K (*T*_p_), respectively (Figure S1a). Accordingly, the previously sluggish crystallization process at temperatures below 842 K (*T*_p_) becomes a rapid one near 842 K (*T*_p_) (Figure S1b). Therefore, the heat treatment temperatures were chosen as 783 and 893 K because these temperatures are expected to result in distinct microstructures owing to large differences in crystallization kinetics. Furthermore, the maximum duration time was determined to be 2 h considering that the microstructure obtained from 2 h annealing remained unchanged after 12 h.^38^

**Figure 1:**
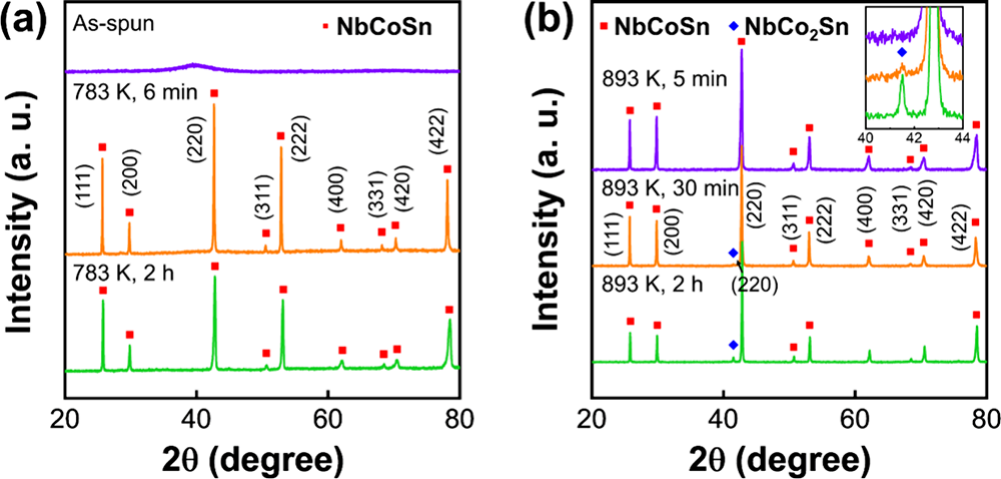
XRD patterns of (a) as-spun NbCo_1.1_Sn, NbCo_1.1_Sn annealed at 783 K for 6 min and 2 h, and (b) NbCo_1.1_Sn annealed at 893 K for 5 min, 30 min, and 2 h.

Figure 1a shows the XRD patterns of the specimens after melt-spinning and annealing at 783 K for 6 min and 2 h. The XRD pattern of the as-spun specimen exhibits only a broad halo peak, indicating a lack of long-range ordering and hence an amorphous structure. After heat treatment at 783 K for 6 min, peaks corresponding to the half-Heusler NbCoSn phase are observed. A single-phase half-Heusler structure is observed after heat treatment for 2 h, and no other phases are detected.

Besides, the XRD patterns of the specimens annealed at 893 K are presented in Figure 1b. Similar to NbCo_1.1_Sn annealed at 783 K, the NbCoSn half-Heusler phase is the primary phase in all three specimens, whereas an additional NbCo_2_Sn Heusler phase is detected in the specimens annealed at 893 K for 30 min and 2 h (inset in Figure 1b).

As the annealing time increases, we observed a reduction in the full width at half-maximum of the NbCoSn (220) peak as the annealing duration increases (Figure S2), indicating the progressive consumption of excessive Co within the NbCo_1+*x*_Sn half-Heusler matrix. The shift in the peak position toward a higher 2theta value (from 42.75 to 42.84) was attributed to the formation of NbCo_2_Sn Heusler precipitates. Calculations based on the (220) peak of NbCoSn demonstrate a reduction in the lattice parameter to 5.966 Å from 5.978 Å after 2 h of annealing at 893 K. Notably, this adjusted value is close to the reported lattice constant of NbCoSn (5.958 Å).^47^ Additionally, NbCo_2_Sn possessed a lattice parameter of 6.148 Å, calculated from the (220) peak of NbCo_2_Sn, which is close to the reported lattice constant of NbCo_2_Sn (6.143 Å).^48^

The mass fraction of NbCo_2_Sn was calculated to be 11% in the specimen annealed for 2h at 893 K, employing scale factor and reference intensity ratio values.^49^ This fraction aligns well with the theoretical prediction for the maximum decomposition from NbCo_1.1_Sn to NbCoSn and NbCo_2_Sn, suggesting the completion of the NbCo_2_Sn formation process after 2 h annealing.

The XRD peak intensities corresponding to the (111) and (200) crystallographic planes in both specimens annealed at 783 and 893 K, respectively, were observed to be different compared to the previously reported patterns of NbCoSn.^50,51^ This can be attributed to the presence of a preferred grain orientation known as texture, which originates from the elongated surface grain.^40^

### 2.2 Isothermal Heat Treatment at 783 K

**Figure 2:**
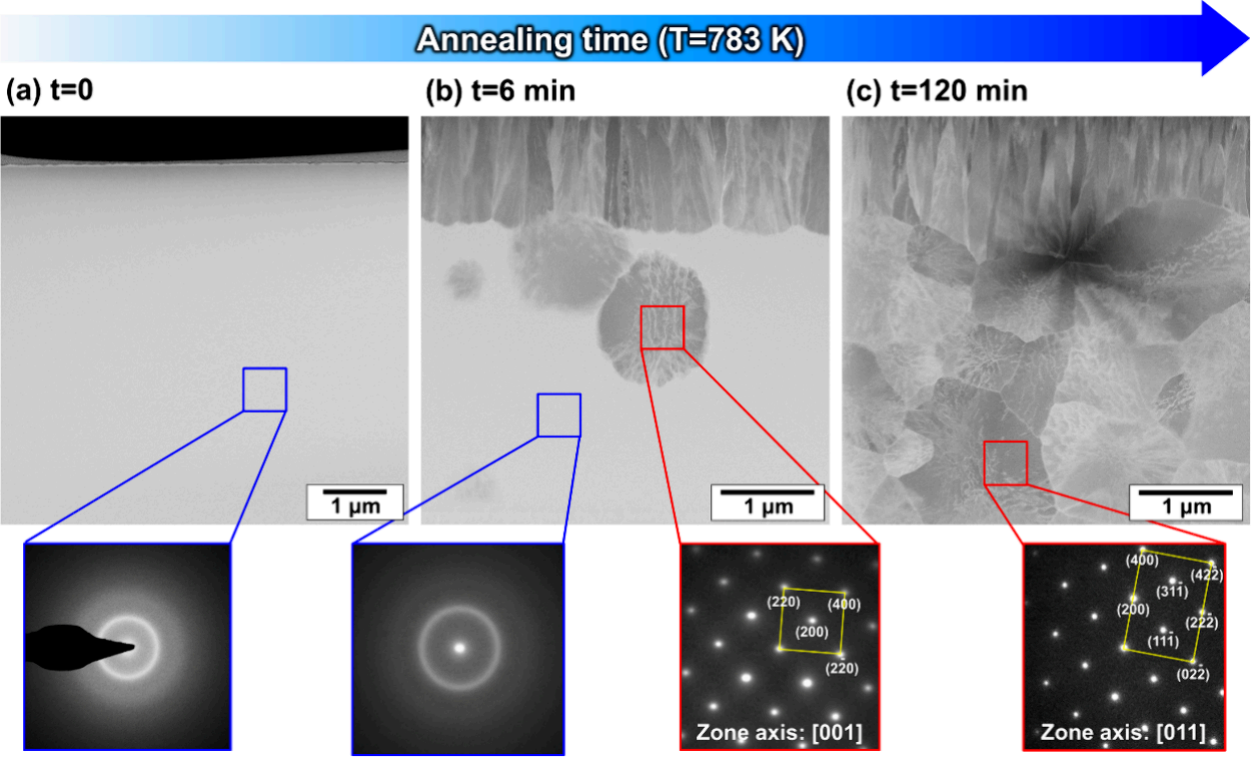
STEM images of (a) as-spun NbCo_1.1_Sn and NbCo_1.1_Sn annealed at 783 K for (b) 6 min and (c) 2 h; insets present the SAED patterns of indicated areas.

Figure 2a shows a STEM image of the as-spun NbCo_1.1_Sn specimen. No crystallites are observed, and the selected area electron diffraction (SAED) pattern shows only halo rings, which is typical for amorphous structures (inset in Figure 2a). Figure 2b shows a specimen annealed at 783 K for 6 min, with a crystalline phase fraction of ∼0.2 based on the DSC results.^38^ Columnar grains are observed near the surface, whereas spherical grains corresponding to the half-Heusler phase are found in the bulk interior, as confirmed using the SAED patterns (red inset in Figure 2b). The homogeneous contrast region was confirmed to be an amorphous region by the halo rings in the SAED pattern, suggesting that a significant portion of the region has remained in an amorphous state without undergoing crystallization (blue inset in Figure 2b). After heat treatment for 2 h, these spherical half-Heusler crystallites grew to an average diameter of 1.1 ± 0.3 μm (Figure 2c). Distinct grain growth has not been observed in NbCo_1.1_Sn annealed at 783 K for 12 h;^38^ therefore, Ostwald ripening does not occur strongly at this temperature.

**Figure 3:**
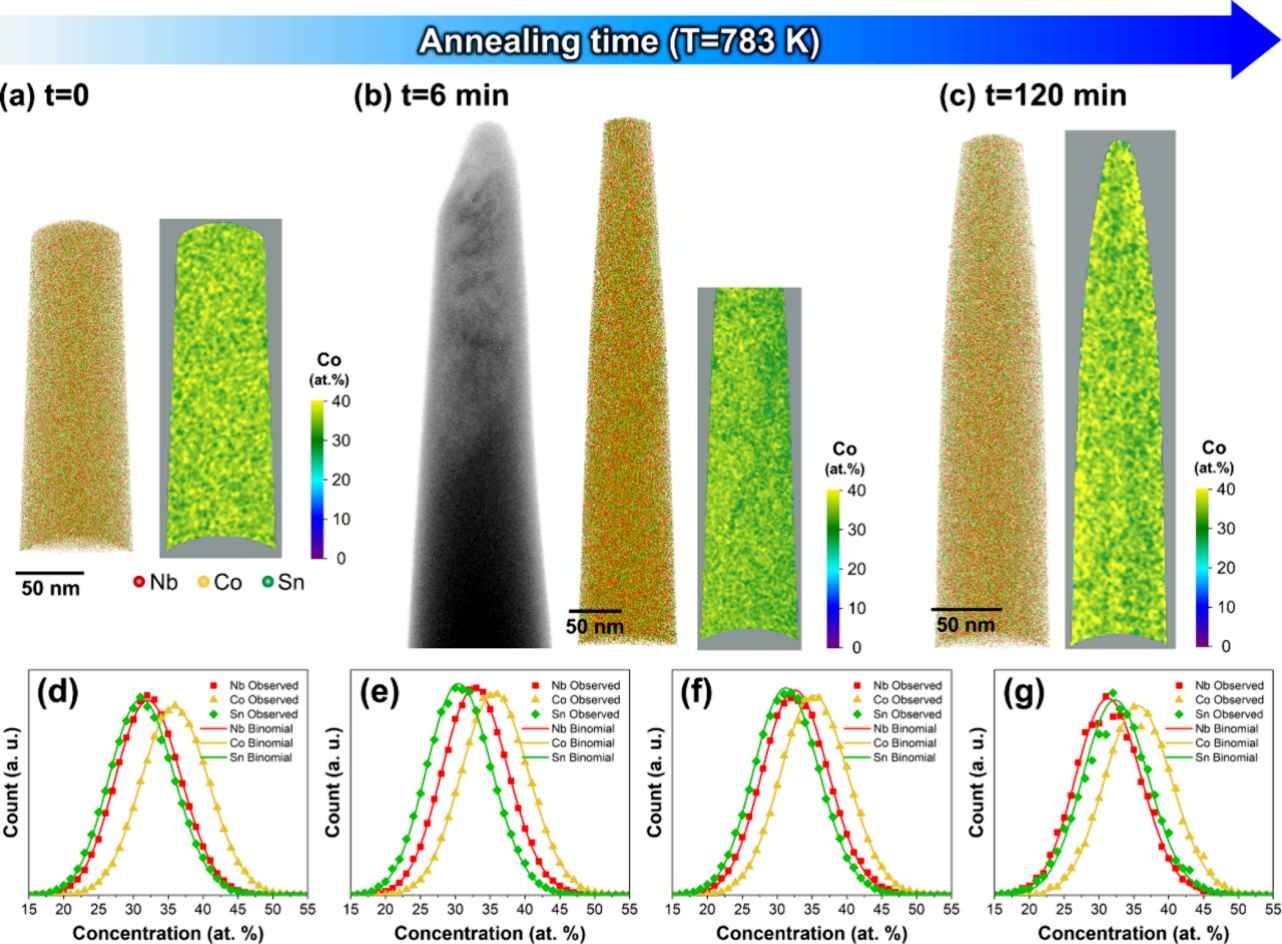
3D atom maps with 2D contour plots of Co composition for (a) as-spun NbCo_1.1_Sn and NbCo_1.1_Sn annealed at 783 K for (b) 6 min and (c) 2 h. Bright field-TEM image is inserted in (b), confirming that the phase boundary was captured in the APT specimen. Frequency distribution analyses for (d) as-spun NbCo_1.1_Sn, (e) amorphous and (f) crystalline regions for NbCo_1.1_Sn annealed at 783 K for 6 min, and (g) NbCo_1.1_Sn annealed at 783 K for 2 h.

The chemical homogeneity of NbCo_1.1_Sn specimens annealed at 783 K was verified using atom probe tomography (APT).^52^Figure 3a shows the 3D atom map and 2D contour plot of Co composition for the as-spun NbCo_1.1_Sn specimen, demonstrating no elemental partitioning in the sample. Figure 3b illustrates the 3D atom map of Nb, Co, and Sn and the 2D contour plot of Co composition for the NbCo_1.1_Sn specimen annealed at 783 K for 6 min, confirming a uniform chemical distribution. The bright-field TEM image of the APT tip validates the presence of the phase boundary between amorphous and crystalline regions in the specimen. Additionally, a pole indicating crystallographic orientation was observed in the top section of the detector map, while homogeneous detection was observed in the bottom part, supporting the presence of an interface (Figure S3). Furthermore, Nb pile-up was observed at the interface and interdendrite region within the crystallite (Figure S3).

To assess the chemical homogeneity, frequency distribution analyses were conducted on the acquired specimens (Figure 3d–g). Binomial distributions of the constituent elements indicate a random distribution; therefore, heterogeneity increases as the difference between the binomial distribution and observed value increases. Since all the observed values were close to the binomial distribution regardless of the annealing time, we concluded that homogeneous chemical distribution was preserved throughout the entire annealing period at 783 K.

### 2.3 Isothermal Heat Treatment at 893 K

**Figure 4:**
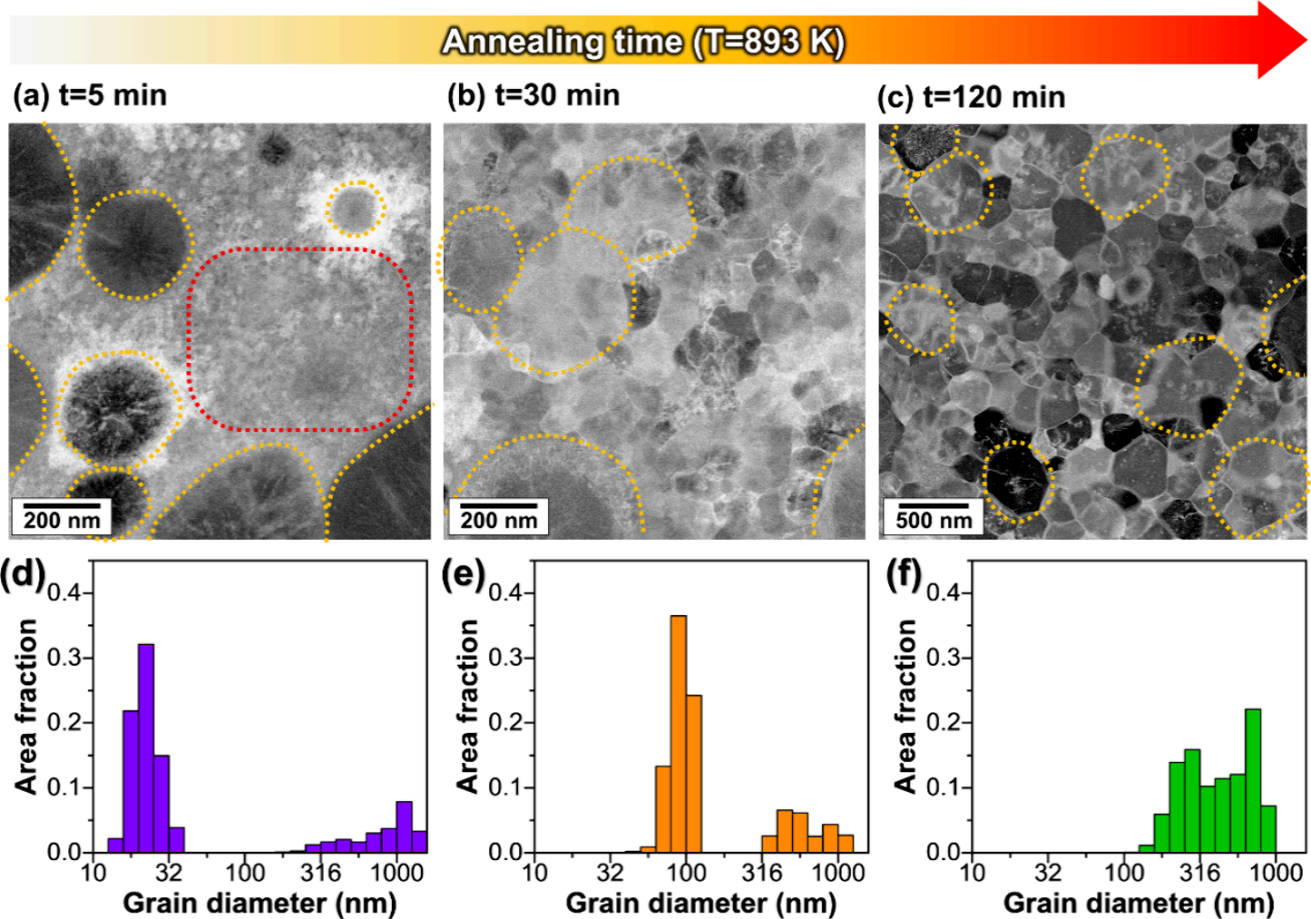
STEM-HAADF images of NbCo_1.1_Sn annealed at 893 K for (a) 5 min, (b) 30 min, and (c) 2 h. Grain size distributions of the specimens annealed at 893 K for (d) 5 min, (e) 30 min, and (f) 2 h.

Figure 4a–c shows the STEM images of a specimen annealed at 893 K for 5, 30, and 2 h, respectively. The specimen annealed for 5 min shows a bimodal size distribution representing coarse spherical grains with a diameter of 500 ± 313 nm (marked with yellow dashed lines in Figure 4a) and small grains with a diameter of 21 ± 4 nm (marked with red dashed lines in Figure 4a). As the annealing time increased, small grains grow to 89 ± 16 nm for 30 min and 219 ± 54 nm for 2 h, whereas coarse grains maintain their sizes (516 ± 176 nm for 30 min and 510 ± 155 nm for 2 h) (Figure 4b,c). As a result, the peaks of the bimodal distribution gradually approach each other and eventually overlap after 2 h of annealing (Figure 4d–f).

**Figure 5:**
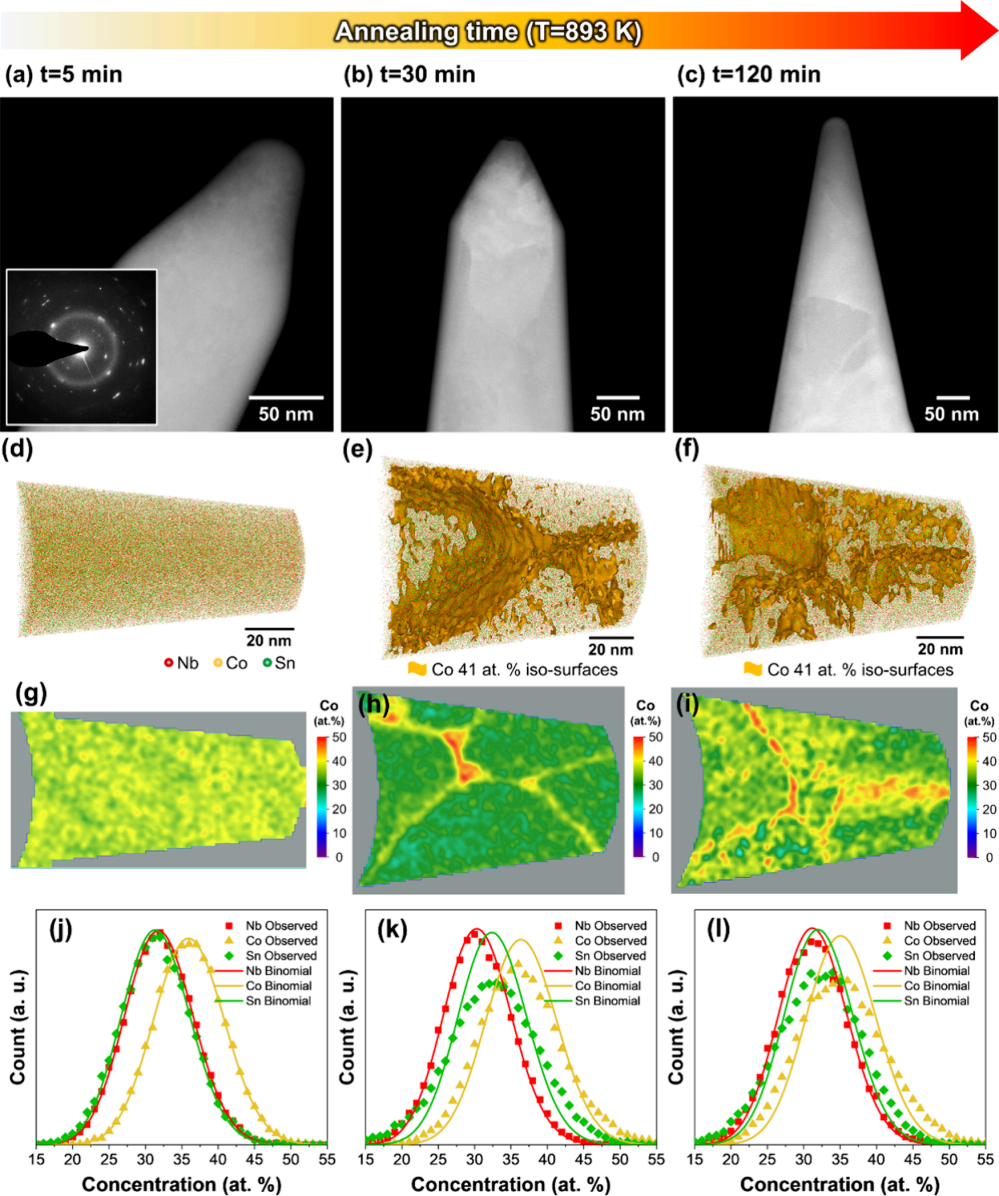
STEM images of APT specimens prepared from the NbCo_1.1_Sn annealed at 893 K for (a) 5 min, (b) 30 min, and (c) 2 h. 3D atom maps for the NbCo_1.1_Sn annealed at 893 K for (d) 5 min, (e) 30 min, and (f) 2 h. 2D contour plots of Co composition for (g) 5 min, (h) 30 min, and (i) 2 h. Frequency distribution analyses for the NbCo_1.1_Sn annealed at 893 K for (j) 5 min, (k) 30 min, and (l) 2 h.

APT was performed to reveal elemental partitioning during heat treatment at 893 K. Figure 5a shows a STEM image of the APT tip for NbCo_1.1_Sn annealed at 893 K for 5 min. Nanometer-sized grains are observed in the prepared APT specimen, which is supported by the SAED pattern, indicating a polycrystalline structure (inset of Figure 5a). We confirmed that the prepared APT specimens from NbCo_1.1_Sn annealed at 893 K for 30 min and 2 h contain multiple grains inside (Figure 5b,c). Figure 5d shows the 3D atom map of NbCo_1.1_Sn annealed at 893 K for 5 min. The composition of the entire volume is 32.03 ± 0.02 at. % Nb, 36.00 ± 0.02 at. % Co, and 31.97 ± 0.02 at. % Sn, which is close to the composition of as-spun NbCo_1.1_Sn determined using ICP-OES (31.5 at. % Nb, 35.6 at. % Co, and 32.9 at. % Sn). Any chemical partitioning was not observed in the reconstructed volume, implying that crystallization occurred without chemical partitioning and yielded half-Heusler nanograins with excess Co composition. However, Co segregation at the grain boundaries is observed for the specimen annealed at 893 K for 30 min and 2 h (Figure 5e,f). The 2D contour plots of Co composition further support that Co segregation occurs after 30 min annealing (Figure 5g–i).

The 1D concentration profiles across the grain boundaries of both specimens display Co enrichment, while Sn and Nb are depleted (Figure S4). The Co segregation volumes exhibit compositions of 29.2 ± 0.1 at. % Nb, 47.6 ± 0.1 at. % Co, and 23.2 ± 0.1 at. % Sn for the specimen annealed at 893 K for 30 min and 28.5 ± 0.2 at. % Nb, 46.7 ± 0.1 at. % Co, and 24.8 ± 0.2 at. % Sn for the specimen annealed at the same temperature but for 2 h. Notably, both compositions closely resemble that of a Heusler compound. Regarding the grain interior, the NbCo_1.1_Sn annealed for 30 min presents composition of 31.5 ± 1.5 at. % Nb, 33.7 ± 1.6 at. % Co, and 34.8 ± 3.0 at. % Sn, while the other specimen exhibits composition of 31.3 ± 1.4 at. % Nb, 33.9 ± 2.0 at. % Co, and 34.7 ± 3.2 at. % Sn. Since the excess Co composition is used to form a Co-rich Heusler phase, the compositions are much closer to a half-Heusler compound.

Moreover, frequency distribution analyses indicate a noticeable deviation between the binomial distribution and the observed value after 30 min of annealing (Figure 5j–l). These results provide further evidence supporting the transition from homogeneous to heterogeneous chemical distribution, which takes place between 5 and 30 min of annealing.

Therefore, amorphous NbCo_1.1_Sn crystallizes into a few-nanometer-sized Co-rich half-Heusler grains without elemental segregation at the early stage of annealing at 893 K. Subsequently, Ostwald ripening of small Co-rich half-Heusler grains and formation of the Heusler phase occur simultaneously. This result is consistent with the XRD results, showing that the Heusler phase was formed after 30 min at 893 K. Consequently, the spherical and disk-shaped Heusler nanoprecipitates are observed within the matrix while Heusler grains are found at the triple boundary of half-Heusler grains.^38^

## 3 Discussion

### 3.1 Grain Size of Annealed Specimens

The NbCo_1.1_Sn specimen annealed at 893 K shows finer grains (219 ± 54 nm and 510 ± 155 nm, Figure 4) than the NbCo_1.1_Sn specimen annealed at 783 K (1.1 ± 0.3 μm, Figure 2). This difference in grain size can be attributed to the differences in crystallization kinetics because crystallization at both temperatures was polymorphic. The temperature region in which the crystallization of an amorphous alloy occurs is above the glass transition temperature, typically 0.5 to 0.65 of the absolute melting temperature.^53,54^ In this region, the nucleation rate increases as the temperature rises, while the growth rate of the nanocrystals increases slowly.^54^ The melting temperature of the NbCo_1.1_Sn alloy was confirmed to be 1467 K using DSC, and the resulting 0.5 and 0.65 absolute melting temperatures are 734 and 954 K, respectively. Thus, our experiments were conducted at the temperature region where the nucleation rate increases dominantly as the temperature rises. As a result, heat treatment at 893 K produced a finer grain size distribution in the specimen than heat treatment at 783 K because of the higher nucleation rate.

### 3.2 Formation of the Heusler Phase

Heat treatment at 783 K resulted in the formation of a Co-rich half-Heusler phase, whereas heat treatment at 893 K resulted in the formation of both half-Heusler and Heusler phases. The differences in the nanostructures resulting from different nanocrystallization processes can be qualitatively discussed.

At 783 K, the amorphous alloy with a composition of NbCo_1.1_Sn crystallized to half-Heusler without elemental redistribution, implying that polymorphic transformation occurred (Figure 3). Subsequently, the Co-rich half-Heusler (NbCo_1.1_Sn) does not decompose to half-Heusler and Heusler phases, presumably owing to limited Co diffusion at 783 K. In contrast, the Co-rich half-Heusler phase crystallizes first from the amorphous matrix through a polymorphic transformation without elemental partitioning at 893 K (Figure 5). Subsequently, the Co-rich half-Heusler compound (NbCo_1.1_Sn) decomposes into half-Heusler and Heusler phases owing to the movement of Co atoms, leading to the formation of nanoprecipitates inside the matrix and Heusler grain through grain boundary diffusion supported by Co segregation at the grain boundaries (Figure 5 and Figure S2).

In XYZ half-Heusler compounds, different grain boundary segregation behaviors of atoms occupying the X-site or Y-site have been observed.^21,22^ For example, in the NbFeSb system,^21^ segregation of Nb was observed, while the VFeSb system exhibited Fe segregation.^55,56^ Therefore, a more comprehensive investigation is required for various half-Heusler compounds to reveal the underlying causes of constituent element segregation and its implications on material properties.

On the other hand, the formation of Heusler nanoprecipitates from the matrix has been widely reported in various half-Heusler compounds including ZrCo_1+*x*_Sb,^56^ NbCo_1+*x*_Sn,^47^ and ZrNi_1+*x*_Sn^57^ half-Heusler compounds. These nanoprecipitates enhance the figure of merit by reducing lattice thermal conductivity through increasing phonon scattering at the interface between the half-Heusler matrix and Heusler nanoprecipitates.

The increased thermal energy and larger grain boundary area of NbCo_1.1_Sn annealed at 893 K, compared to those annealed at 783 K, may facilitate the diffusion of Co atoms. This diffusion process contributes to the Co diffusion and formation of the Heusler phase, ultimately increasing the phonon scattering. (Figures 5).

### 3.3 Microstructural Evolution during the Annealing

**Figure 6:**
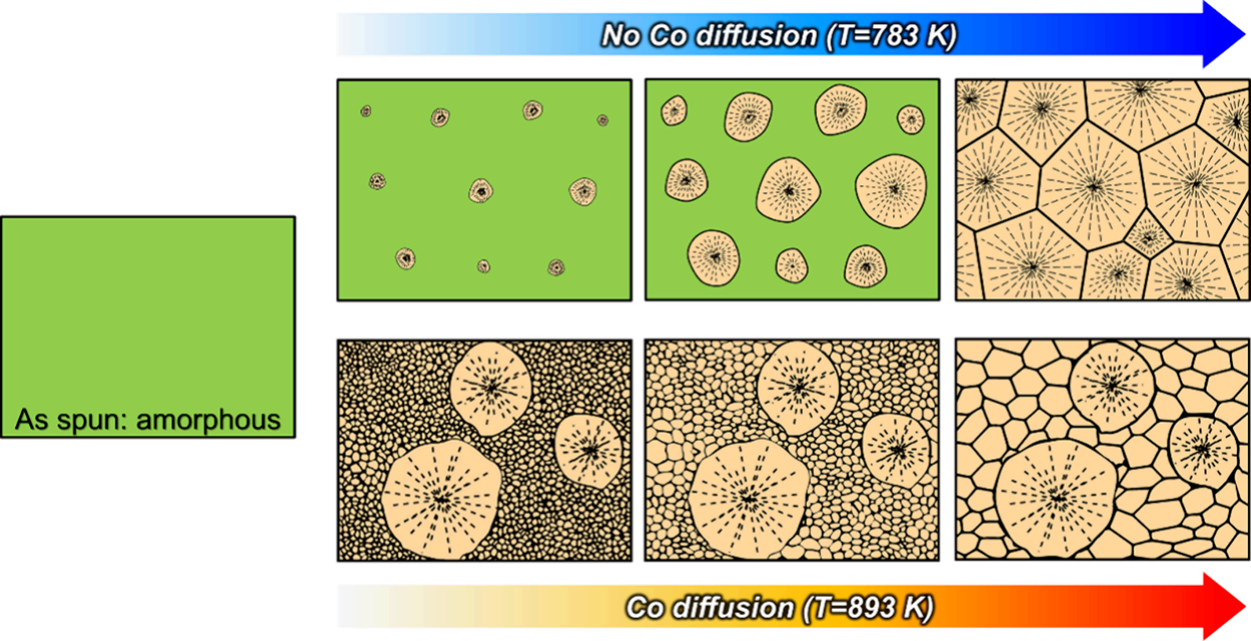
Schematic of the microstructural evolution of the amorphous NbCo_1.1_Sn alloy during heat treatment at 783 and 893 K.

A bimodal grain structure is observed in NbCo_1.1_Sn annealed at 893 K (Figure 4). The crystallization of this specimen is completed before the temperature reached 893 K.^38^ When heated to 893 K, a few spherical NbCo_1.1_Sn half-Heusler crystallites nucleate and grow into coarse grains below 842 K (*T*_p_), whereas small grains are formed at a high nucleation rate at 842 K (*T*_p_). Subsequently, Ostwald ripening causes these small grains to grow (Figure 4), where Co partitioning occurs during heat treatment at 893 K for 2 h (Figure 5). In contrast, several nuclei grow owing to the sluggish nucleation rate and impinge on each other, resulting in a coarse-grained structure after heat treatment at 783 K for 2 h (Figure 2). The microstructural evolution of the amorphous NbCo_1.1_Sn alloy during heat treatment at 783 and 893 K is summarized in Figure 6.

### 3.4 Impact on Lattice Thermal Conductivity

To understand the lower lattice thermal conductivity of the NbCo_1.1_Sn annealed at 893 K than that annealed at 783 K, the Debye–Callaway model is used to calculate the theoretical lattice thermal conductivities of both specimens at room temperature.^58,59^ The detailed explanations of the equations associated with each scattering process can be found in the Supporting Information.^60−63^

**Figure 7:**
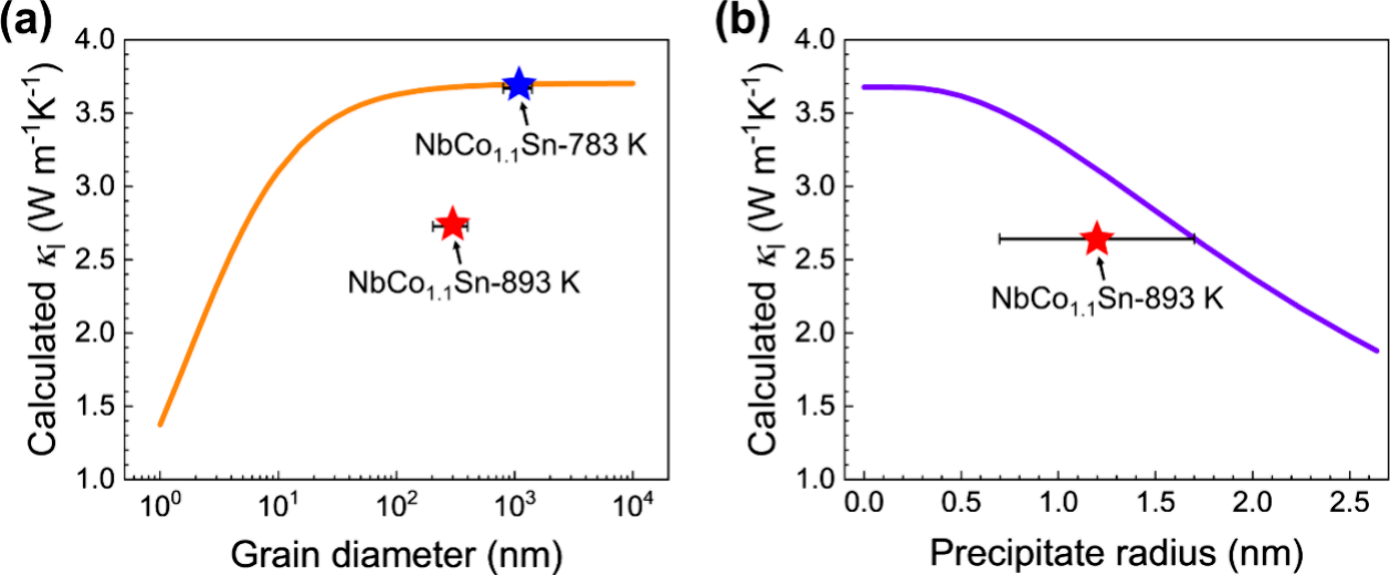
Calculated and measured lattice thermal conductivities depending on the (a) grain diameter and (b) precipitate radius at 298 K. The orange and purple curves represent the calculated lattice thermal conductivities using the Debye–Callaway model, while the star-shaped data points display the measured values of lattice thermal conductivities.

Figure 7a shows the calculated lattice thermal conductivity depending on the grain size. The results clearly demonstrate that a significant reduction in lattice thermal conductivity occurs when the grain diameter is below 100 nm. In contrast, the lattice thermal conductivity remains relatively constant in the range where the grain diameter exceeds 100 nm. For the half-Heusler compounds, He et al. demonstrated that ∼10 nm grain size is required to obtain a significant reduction in lattice thermal conductivity from grain boundary scattering.^30,64^ In addition, first-principles calculation proved that the majority of phonons responsible for heat conduction possess mean free paths smaller than a 100 nm scale.^18^ These previous results are consistent with our results shown in Figure 7a. The experimental lattice thermal conductivities of NbCo_1.1_Sn annealed for 2 h at 783 and 893 K at room temperature were 3.69 and 2.74 W/mK, respectively.^38^ Considering that average grain diameters of both specimens exceed 100 nm, the disparity in lattice thermal conductivities cannot be attributed to differences in grain size.

Next, we investigated the effect of Heusler nanoprecipitates on lattice thermal conductivity. Figure 7b displays the calculated lattice thermal conductivity depending on the radius of nanoprecipitates. We employed the previously reported value of 1.3 × 10^24^ m^–3^ for the number density of precipitates^38^ and increased the radius of precipitates within the volume fraction of precipitates that remained below 10% (with a radius of 2.64 nm) considering the amount of excess Co composition. Unlike grain diameter, the calculated lattice thermal conductivity significantly decreases as the precipitate radius increases within a fixed number density of precipitates.

The average grain diameters of NbCo_1.1_Sn specimens annealed at 783 and 893 K were approximately 300 and 1100 nm, respectively. The difference in grain size between the specimens does not affect the lattice thermal conductivity since the calculated lattice thermal conductivity remains constant at 3.7 W m^–1^ K^–1^ for the grain size above 300 nm (Figure 7a). Therefore, the decreased lattice thermal conductivity in the specimen annealed at 893 K can be attributed to the formation of Heusler nanoprecipitates as a result of Co diffusion. Including the nanoprecipitate term in the Debye–Callaway model (Table S1), the lattice thermal conductivity was found to be 2.74 W m^–1^ K^–1^ when the precipitate radius was 1.6 nm. This radius was close to the experimentally measured one (1.2 ± 0.5 nm),^38^ confirming that precipitate scattering is in charge of reduction in lattice thermal conductivity.

## 4 Conclusions

In this study, we elucidated the formation of two different nanostructures upon the heat treatment of an amorphous NbCo_1.1_Sn alloy at 783 and 893 K using APT and TEM. The specimen annealed at 893 K showed finer grains than the specimen annealed at 783 K because of the higher nucleation rate at 893 K than at 783 K. The specimen annealed at 893 K contained both half-Heusler and Heusler phases, whereas the specimen heat-treated at 783 K contained only the half-Heusler phase owing to the limited kinetics of Co atoms. Furthermore, the Debye–Callaway model supports that the reduction in lattice thermal conductivity is attributed to the formation of Heusler nanoprecipitates rather than a finer grain size. Understanding the crystallization behavior of amorphous NbCo_1.1_Sn gives the insight to tailor the nanostructure for enhanced thermoelectric properties. For instance, controlling the size of nanoprecipitates or engineering grain boundary segregation/phase will be the subject of future research.

## 5 Materials and Methods

### 5.1 Sample Preparation

An ingot of NbCo_1.1_Sn with the target composition (at. %) was prepared via vacuum arc melting using elemental granule with high purity (Nb and Co > 99.95%; Sn > 99.99%) under an Ar (>99.999%) gas atmosphere. An excess of Sn (∼2 wt %) was added to compensate for its loss due to evaporation. The ingot was remelted five times to ensure chemical homogeneity. Subsequently, the ingot was cut into a disk shape with 12.5 mm diameter and 8 mm thickness. To fabricate amorphous ribbons, the melt-spinning technique was applied. Prior to heating the alloy, the pressure was set to 2 × 10^–3^ Torr using a rotary pump. Following this, the chamber pressure was regulated up to 5 × 10^2^ Torr by purging with Ar gas. This process should be repeated three times to establish an inert chamber atmosphere. Subsequent to this, the alloy within a quartz tube having an inner diameter of 13 mm and a thickness of 1.5 mm was subjected to heating, reaching approximately 1500 K, resulting in a molten alloy. A pressure of 3 × 10^–2^ MPa was applied to eject molten alloy onto a rotating Cu wheel (1.05 × 10^2^ mm in diameter) at a speed of 40 m/s. Consequently, an amorphous ribbon (∼20 μm thick and ∼500 μm wide) with an ingot composition was obtained. The heating rate of both specimens was set to 40 K/min in accordance with the DSC experimental condition.

### 5.2 Characterization

The crystallization temperature was measured by differential scanning calorimetry (DSC; PerkinElmer PYRIS Diamond) at a heating rate of 40 K/min. Inductively coupled plasma-optical emission spectrometry (ICP-OES; Thermo Fisher Scientific ICAP 6500) was used to determine the average chemical composition of the as-spun ribbons. Structural characterization was performed using X-ray diffraction (XRD; (RIGAKU SmartLab)) with Cu Kα radiation. A dual-beam focused ion beam/scanning electron microscopy platform (FEI Helios 450 F1) was used to prepare specimens for APT and TEM, as described in refs (65) and (66) Site-specific APT specimens were attached to a TEM half grid to perform correlative TEM prior to APT.^67,68^ Bright-field TEM (Thermo Fisher Talos F200X operated at 200 kV) was used to observe the APT specimen prepared from NbCo_1.1_Sn annealed at 783 K for 6 min. Scanning transmission electron microscopy (STEM; Thermo Fisher Talos F200X operated at 200 kV) was used to conduct high-angle annular dark field (HAADF) imaging. APT analyses were performed using a local electrode atom probe (LEAP 4000X HR, CAMECA Instrument) in the pulsed laser mode at 50 K with the laser pulse energy and frequency of 50 pJ and 125 kHz, respectively. The overlapping peaks of Co^+^ and Sn^2+^ ions at the mass-to-charge ratio of 59 Da resulted in the uncertainty of Co and Sn concentration by around 1.0 at %. Data reconstruction and analyses were performed using IVAS 3.8.8 software provided by CAMECA instruments.

## Supporting Information Available

More information and details about the description of the Debye–Callaway model and used parameter in this study, DSC curve of the as-spun NbCo_1.1_Sn specimen, enlarged XRD patterns of NbCo_1.1_Sn annealed at 893 K, 3D atom map with 2D density contour plot for the specimen annealed at 783 K for 6 min, and 2D density contour plots with 1D concentration profiles for NbCo_1.1_Sn annealed at 893 K (PDF)
