# Supplementary material for: Effect of Heat Treatment Temperature on the Crystallization Behavior and Microstructural Evolution of Amorphous NbCo1.1Sn
Source: ACS Appl Mater Interfaces. 2023 Sep 22;15(39):46064–73. doi: 10.1021/acsami.3c10298 (PMC10561143; doi:10.1021/acsami.3c10298)
Supplement: Supplementary file 1 — am3c10298_si_001.pdf [file am3c10298_si_001.pdf]

Supporting Information

Effect of heat treatment temperature on the crystallization behavior and microstructural evolution of amorphous $NbCo_{1.1}Sn$

Chanwon Jung [a,b,*], Siyuan Zhang [b], Kyuseon Jang [a], Ningyan Cheng [b], Christina Scheu [b], Seong-Hoon Yi [c,*], Pyuck-Pa Choi [a,*]

[a] Department of Materials Science and Engineering, Korea Advanced Institute of Science and Technology (KAIST), 291 Daehak-ro, Yuseong-gu, Daejeon 34141, Republic of Korea

[b] Max-Planck-Institut für Eisenforschung, Max-Planck-Straße 1, 40237 Düsseldorf, Germany

[c] Department of Materials Science and Metallurgical Engineering, Kyungpook National University, 80 Daehakro, Daegu 41566, Republic of Korea

* Corresponding Authors: c.jung@mpie.de, p.choi@kaist.ac.kr; yish@knu.ac.kr

Keywords

Amorphous, crystallization, half-Heusler compounds, diffusion, atom probe tomography

Debye-Callaway model

Debye–Callaway model is used to calculate theoretical lattice thermal conductivities of both specimens at room temperature, as below [1,2].

$$\kappa_l = \frac{k_B}{2\pi^2 v}\left(\frac{k_B T}{\hbar}\right)^3 \int_0^{\frac{\theta_D}{T}} \tau_{tot}(x)\frac{x^4 e^x}{(e^x-1)^2}dx \qquad (1)$$

Here, $k_B, v,$ and θ_D represent the Boltzmann constant, phonon velocity, and Debye temperature, respectively. x is defined as $\hbar\omega/k_B T$, where ω is the phonon frequency. $\tau_{tot}(x)$ is the phonon relaxation time, which is determined by Matthiessen's rule as described by equation (2) [3].

$$\tau_{tot}{}^{-1} = \sum_i \tau_i{}^{-1} = \tau_U{}^{-1} + \tau_{pe}{}^{-1} + \tau_{GB}{}^{-1} + \tau_{NPs}{}^{-1} \qquad (2)$$

where τ_U, τ_{pe}, τ_{GB}, and τ_{NPs} are the relaxation times of the phonon–phonon Umklapp scattering, phonon–free electron scattering, phonon–grain boundary scattering and phonon–nanoprecipitate scattering, respectively.

For the Umklapp scattering, τ_U is empirically described as follows [3]:

$$\tau_U{}^{-1} = \frac{\hbar\gamma^2\omega^2 T}{M v^2 \theta_D} exp\left(-\frac{\theta_D}{3T}\right) \qquad (3)$$

where γ is Grueneisen parameter, which is highly dependent on the anharmonicity of the bonding and M is average mass.

For the phonon-free electron scattering, τ_{pe} is described by the following equation under the high carrier concentration [3,4]:

$$\tau_{pe}{}^{-1} = \frac{E_{def}^2 m_*^2 \omega}{2\pi\hbar^3 \rho v_l} \qquad (4)$$

where E_{def}^2 and v_l are the deformation potential and longitudinal sound velocity, respectively.

The grain boundary scattering in polycrystalline materials is estimated as below [3]:

$$\tau_{GB}{}^{-1} = \frac{v}{d} \qquad (5)$$

Here, d is the grain diameter.

For phonon–nanoprecipitate scattering, τ_{NPs} can be described by the following equation [5,6]:

$$\tau_{NPs}{}^{-1} = vN_p\left[(2\pi R^2)^{-1} + \left(\frac{4}{9}\pi R^2\left(\frac{\Delta\rho}{\rho}\right)^2\left(\frac{\omega R}{v}\right)^4\right)^{-1}\right]^{-1} \qquad (6)$$

where $N_p, R, \rho,$ and $\Delta\rho$ are the number density of the precipitates, average radius, matrix density, and density difference between the matrix and precipitates, respectively. The parameters used to calculate the lattice thermal conductivities are listed in Table S1.

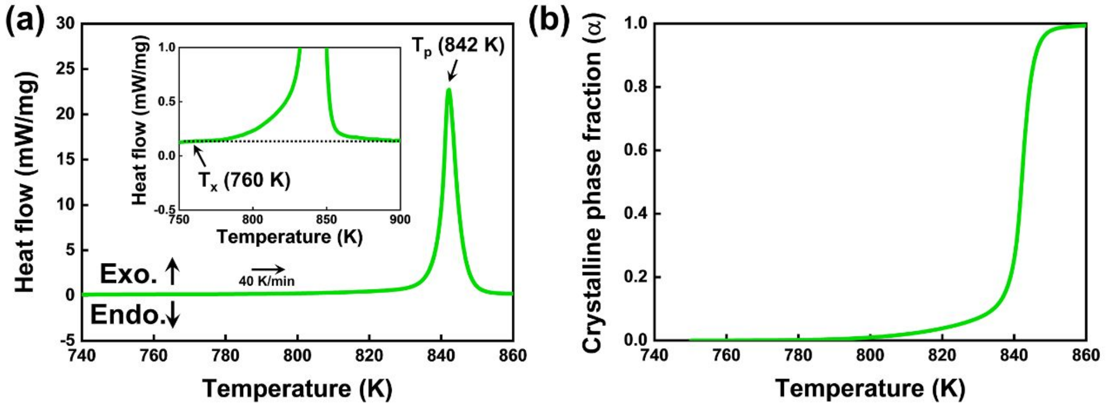

Figure S1. (a) DSC curve recorded at a heating rate of 40 K/min. (b) Volume fraction of the crystalline phase as a function of temperature. The crystalline phase fraction as a function of temperature is calculated as follows: $\boldsymbol{\alpha} = \int_{T_0}^{T}\left(dH_c/dT\right)dT \Big/ \int_{T_0}^{T_\infty}\left(dH_c/dT\right)dT$, where T_0 and T_∞ are the temperatures at which crystallization begins and ends, respectively, and dH_c/dT is heat flow rate.

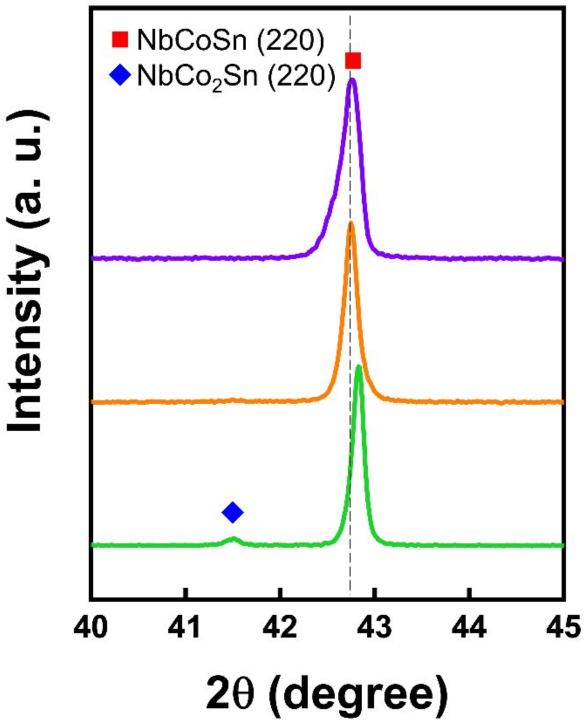

Figure S2. Enlarged XRD patterns of $NbCo_{1.1}Sn$ annealed at 893 K for 5 min (purple), 30 min (orange), and 2 h (green).

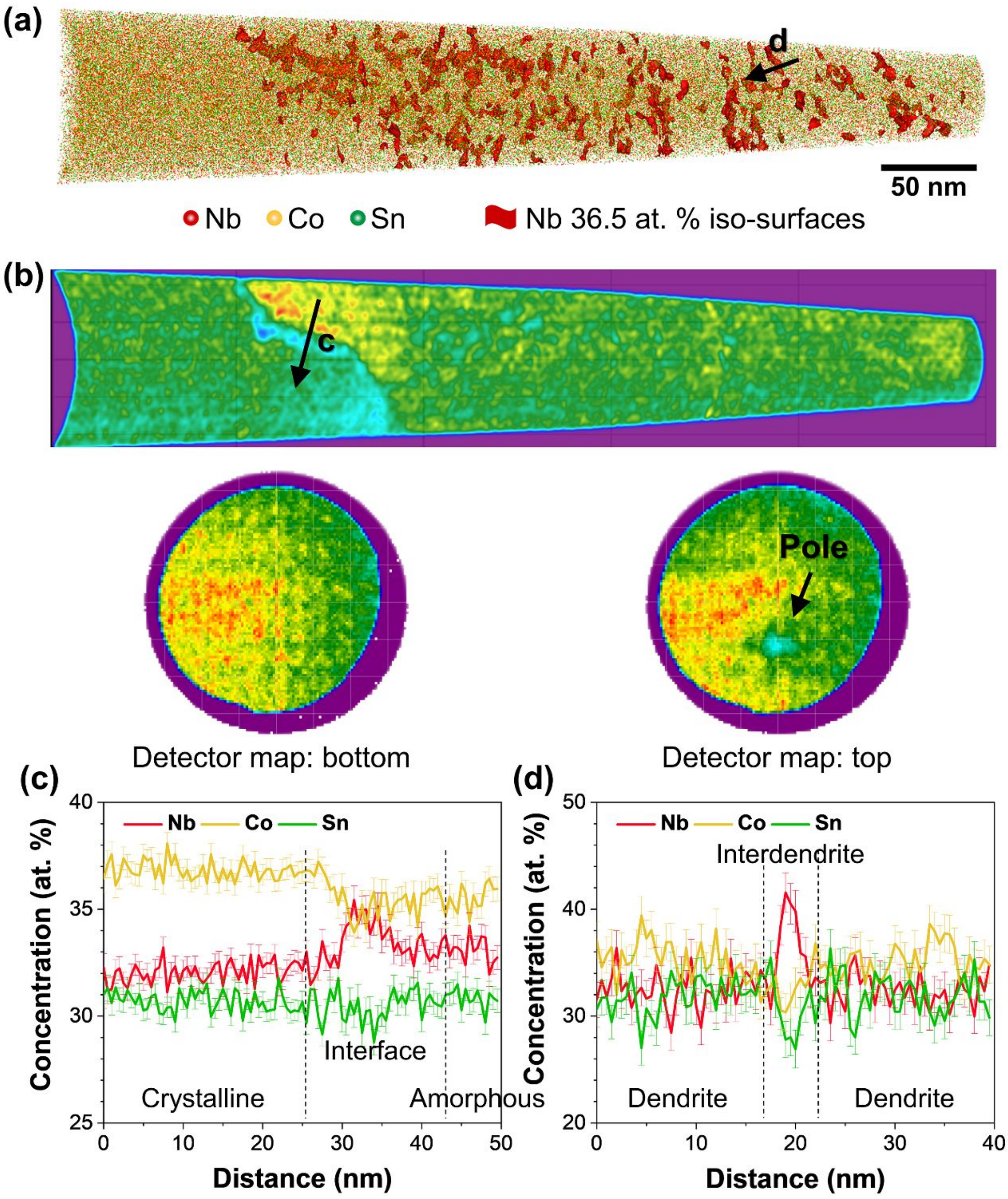

Figure S3. (a) 3D atom map with (b) 2D density contour plot for $NbCo_{1.1}Sn$ annealed at 783 K for 6 min. 1D concentration profiles across the (c) phase boundary and (d) interdendrite region.

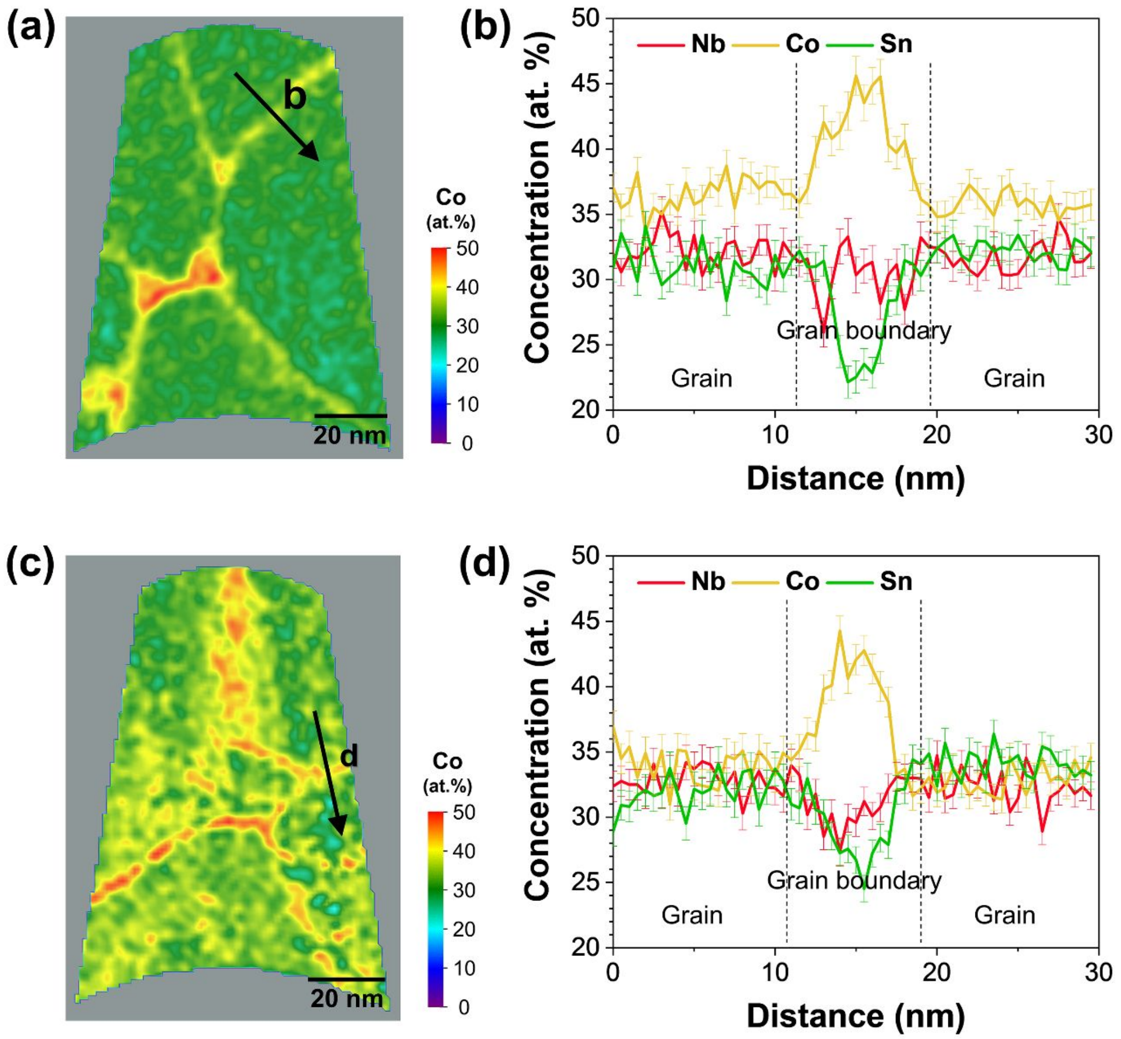

Figure S4. 2D density contour plots for $NbCo_{1.1}Sn$ annealed at 893 K for (a) 30 min and (b) 2 h. 1D concentration profiles across the grain boundary from $NbCo_{1.1}Sn$ annealed at 893 K for (b) 30 min and (d) 2 h.

Table S1. Parameters used to calculate the lattice thermal conductivities of NbCo1.1Sn annealed at 783 and 893 K for 2h based on the Debye–Callaway model.

Parameters	$NbCo_{1.1}Sn$ annealed at 783 K, 2h	$NbCo_{1.1}Sn$ annealed at 893 K, 2h	Comment
θ_D (K)	361		[7]
v_s (m/s)	3141		[7]
v_l (m/s)	4956		[8]
E_{def}(eV)	4.3		Fitted
m_d^*	6.9 m_e		[9]
γ	1.2		[8]
d (nm)	1100	300	Measured
N_p (10^{21} m^{-3})	-	1.3×10^3	[10]
R (nm)	-	1.6	[10]
ρ (g/cm^{-3})	8.35	8.35	[11]
$\Delta\rho$ (g/cm^{-3})	-	-0.95	[12]
Calculated κ_l (W m^{-1} K^{-1})	3.70	2.74	Calculated
Measured κ_l (W m^{-1} K^{-1})	3.69	2.74	[10]

References

[1] J. Callaway, Model for lattice thermal conductivity at low temperatures, Phys. Rev. 113 (1959) 1046.

[2] J. Callaway, H.C. von Baeyer, Effect of Point Imperfections on Lattice Thermal Conductivity, Phys. Rev. 120 (1960) 1149–1154. https://doi.org/10.1103/PhysRev.120.1149.

[3] H. Xie, H. Wang, Y. Pei, C. Fu, X. Liu, G.J. Snyder, X. Zhao, T. Zhu, Beneficial contribution of alloy disorder to electron and phonon transport in half-heusler thermoelectric materials, Adv. Funct. Mater. 23 (2013) 5123–5130. https://doi.org/10.1002/adfm.201300663.

[4] Parrott, J. E. Heat Conduction Mechanisms in Semiconducting Materials. (1979).

[5] N. Mingo, D. Hauser, N.P. Kobayashi, M. Plissonnier, A. Shakouri, "Nanoparticle-in-Alloy" approach to efficient thermoelectrics: silicides in SiGe, Nano Lett. 9 (2009) 711–715.

[6] Z. Chen, Z. Jian, W. Li, Y. Chang, B. Ge, R. Hanus, J. Yang, Y. Chen, M. Huang, G.J. Snyder, Lattice dislocations enhancing thermoelectric PbTe in addition to band convergence, Adv. Mater. 29 (2017) 1606768.

[7] D.A. Ferluccio, R.I. Smith, J. Buckman, J.-W.G. Bos, Impact of Nb vacancies and p-type doping of the NbCoSn–NbCoSb half-Heusler thermoelectrics, Phys. Chem. Chem. Phys. 20 (2018) 3979–3987.

[8] R. Yan, W. Xie, B. Balke, G. Chen, A. Weidenkaff, Realizing p-type NbCoSn

half-Heusler compounds with enhanced thermoelectric performance via Sc substitution, Sci. Technol. Adv. Mater. 21 (2020) 122–130.

[9] Q. Wang, J. Huang, C. Wang, P. Luo, Z. Li, R. Liu, Q. Ma, J. Luo, Enhancement of Thermoelectric Properties in n-type NbCoSn Half-Heusler Compounds via Ta Alloying, ACS Appl. Energy Mater. 4 (2021) 12458–12465.

[10] C. Jung, B. Dutta, P. Dey, S. Jeon, S. Han, H.-M. Lee, J.-S. Park, S.-H. Yi, P.-P. Choi, Tailoring nanostructured NbCoSn-based thermoelectric materials via crystallization of an amorphous precursor, Nano Energy. 80 (2021) 105518. https://doi.org/10.1016/j.nanoen.2020.105518.

[11] R. He, L. Huang, Y. Wang, G. Samsonidze, B. Kozinsky, Q. Zhang, Z. Ren, Enhanced thermoelectric properties of n-type NbCoSn half-Heusler by improving phase purity, APL Mater. 4 (2016). https://doi.org/10.1063/1.4952994.

[12] https://www.ctcms.nist.gov/~knc6/jsmol/JVASP-15412.html
